# Machine Learning to Differentiate Between Positive and Negative Emotions Using Pupil Diameter

**DOI:** 10.3389/fpsyg.2015.01921

**Published:** 2015-12-22

**Authors:** Areej Babiker, Ibrahima Faye, Kristin Prehn, Aamir Malik

**Affiliations:** ^1^Center for Intelligent Signal and Imaging Research, Universiti Teknologi PETRONASBandar Seri Iskandar, Malaysia; ^2^Department of Electrical and Electronic Engineering, Universiti Teknologi PETRONASBandar Seri Iskandar, Malaysia; ^3^Department of Fundamental and Applied Sciences, Universiti Teknologi PETRONASBandar Seri Iskandar, Malaysia; ^4^Department of Neurology and NeuroCure Clinical Research Center, Charité Universitätsmedizin BerlinBerlin, Germany

**Keywords:** pupillometry, emotion recognition, classification, k-nearest neighbor algorithm, sensitivity analysis

## Abstract

Pupil diameter (PD) has been suggested as a reliable parameter for identifying an individual’s emotional state. In this paper, we introduce a learning machine technique to detect and differentiate between positive and negative emotions. We presented 30 participants with positive and negative sound stimuli and recorded pupillary responses. The results showed a significant increase in pupil dilation during the processing of negative and positive sound stimuli with greater increase for negative stimuli. We also found a more sustained dilation for negative compared to positive stimuli at the end of the trial, which was utilized to differentiate between positive and negative emotions using a machine learning approach which gave an accuracy of 96.5% with sensitivity of 97.93% and specificity of 98%. The obtained results were validated using another dataset designed for a different study and which was recorded while 30 participants processed word pairs with positive and negative emotions.

## Introduction

Emotions have a significant impact on perception, decision making, action generation, as well as action execution and control (e.g., Surakka and Sams, 1999; Zhu and Thagard, 2002). Processes such as learning, attention, perception, and memory are affected by emotions. Recognizing emotional expressions is important for the development of Human computer interaction (HCI) systems. Because of the manifoldness and complexity of emotional expressions, much research has been conducted to understand and explain the mechanisms involved in emotion recognition. This is driven by the huge amount of promising usages and benefits such systems might have. In the field of social and clinical psychology, emotion detection systems could help to diagnose psychological disorders including fatig, stress, and depression at their very early stages (Kulkarni et al., 2009). In entertainment and video game industry, artificial characters who interact with the player have been developed (Maes, 1995). Moreover, in applications where computers play a social role such as companion or instructor, the functionality of the system could be improved dramatically if the system could automatically recognize a user’s emotions and take appropriate action. Emotion detection has been further applied to learning process through feedback which increases student’s motivation and interaction with the learning environment and thus maximizes learning outcomes (Pampouchidou, 2011, Unpublished,). Automatic face tracking expression analysis has already been integrated in automatic animated tutoring systems (Bartlett et al., 2003). Other possible applications such as stress classification have also gained intensive attention (Pedrotti et al., 2014). This paper is an attempt to differentiate positive and negative emotions.

Emotions are recognized in different ways: (1) visually (frosm facial expressions displayed on pictures and in videos), (2) by changes in signals of the autonomic nervous system (ANS), and (3) acoustically (from the human voice; Lisetti, 2002). This paper addresses emotion detection based on ANS signals, and specifically, using Pupil Diameter (PD). The pupil is the black hole in the middle of the iris that regulates light entrance to the retina. It is regulated by the ANS that consists of three divisions: the sympathetic, the parasympathetic, and the enteric system (Partala and Surakka, 2003; Ren et al., 2011). The parasympathetic and sympathetic divisions of the ANS govern two sets of muscles in the iris called the sphincter and the dilator, which are both responsible for changes in PD. However, Bradley et al. (2008) supported the hypothesis that pupillary changes are mainly associated with sympathetic activity. Changes in PD have been proven to be optimal in measuring human emotion although differences in luminance between stimuli may have some influence in an uncontrolled environment (e.g., Geangu et al., 2011). Moreover, measurement of the pupil size has important advantages over other physiological signals, such as heart rate and skin conductance, because it is less affected by body gestures. Besides this, it depends solely on the ANS that is largely unconscious and difficult to control voluntarily. Changes of the PD occur with a short latency and can be recorded by a camera without attaching any sensors which makes data acquisition more convenient than the recording of skin conductance or heart rate (see Partala and Surakka, 2003; Adolphs, 2006; Wang, 2010; Lanata et al., 2011). The eye tracking system or the technology necessary for accurate measurement is relatively cheaper and simpler to use compared to the technologies for measuring other signals (e.g., EEG).

Previous findings and research showed that pupil dilation indicates cognitive load as well as emotions and arousal (e.g., Hess, 1972; Bradley et al., 2008; Wang, 2010). A study conducted by Hess and Polt (1960), was the beginning of pupillary responses research. It showed that PD is related to “feeling tone” or emotions caused by picture viewing and was followed by several studies confirming pupillary dilation in response to emotions (Woodmansee, 1967; Janisse, 1974; Andreassi, 2000; Lisetti and Nasoz, 2004; Valverde et al., 2010). Majority of recent studies have provided evidence for greater dilation in PD during the processing of both positive and negative compared to neutral stimuli (Partala and Surakka, 2003; Bradley et al., 2008).

Subjectively experienced emotional states can be characterized by the dimensions valence and arousal (Wundt, 1924; for a review, see Feldman Barrett and Russell, 1999). Valence represents the hedonic tone of an emotion (i.e., pleasure – displeasure), whereas emotional arousal refers to the energy level of the emotion (i.e., the psycho-physiological level of activation). In addition to this dimensional approach, a set of different qualitative emotional states has been suggested comprising happiness, surprise, fear, anger, and disgust (often referred to as basic emotions; Ekman and Friesen, 2003, for critical discussion see also Barrett, 1998).

Measurement of emotion is affected by one’s emotional status, e.g., subjective experience, physiology, culture, and behavior. Here, we applied self-report measurement that is represented in Positive and Negative Affect Schedule – Expanded form (PANAS-X) model (Watson and Clark, 1999).

Machine learning techniques are consisted of a group of effective statistical methods in the field of pattern recognition, in particular, with high-dimensional problems (Trabelsi and Frasson, 2010; Kragel and Labar, 2013; Chang et al., 2015). One of the simplest techniques of these groups are the nearest neighbor. Their rule identifies the class of unknown data point based on its nearest neighbor whose class is already known (Bhatia and Vandana, 2010). kNN has been used extensively in emotion recognition researches either individually or combined with other machine learning techniques (Murugappan et al., 2010; Meftah et al., 2012; Hatamikia et al., 2014). It has several advantages over other traditional approaches such as simple implementation, wide range of parameter’s choice, and model freeness. In kNN, the data is divided into a training set (labeled examples) and a testing set (unlabeled examples). A new instant (unlabeled example) is classified based on its similarity with the examples in the training set.

In the present study, we investigated whether pupillary responses during the processing of positive and negative stimuli differ in behavior and whether this difference can be used to classify emotions into positive and negative ones using a machine learning approach. Two datasets were used in this study. The first dataset, used 20 sound stimuli that were divided into 10 negative sounds and 10 positive sounds (IADS; Bradley and Lang, 1999). The second dataset was collected by Prehn et al. (2011), and used to validate the model. The authors presented participants with word pairs with emotional content and participants decided whether word pairs corresponded in both their emotional and conceptual relations.

## Materials and Methods

To investigate whether there is significant difference in pupil dilation while processing positive and negative stimuli that can be applied to classify pupillary responses using a machine learning approach, we conducted an experiment in which participants heard emotional sounds while their PD was measured. The experimental procedures were approved by Universiti Teknologi PETRONAS Ethical Committee.

### Participants

Thirty healthy subjects (17 males) with normal and corrected-to-normal vision participated in the study. The participants were university students, with mean age of 24.56 years (*SD* = 2.87). The participants were not affected by any medication that could influence pupillary response. A briefing about the experiment was given and a consent form was signed by each participant.

Another dataset (Prehn et al., 2011) was used to validate and confirm the result obtained from the first dataset. In the second study 30 healthy subjects (11 males) participated with mean age of 23.93 years (*SD* = 4.34). Participants were native German speakers and did not take any medication that could influence pupillary response. They also gave written consent and received either course credit or payment (20 Euro) for their participation. Throughout the present study, datasets will be referred to as first dataset and second dataset, respectively.

### Stimuli

Stimulations that are used to trigger emotions are of three types: visual, audio, and audio–visual. In this experiment, audio stimulation was used. To ensure the spontaneity and occurrence of desired emotional states, a strong effective stimulation was used. The twenty sound stimuli were divided into 10 negative sounds and 10 positive sounds and differed significantly in valence [*M* = 4.5, *SD* = 1.78, *t*(9) = -9.32, *p* < 0.001], and in arousal [*M* = 5.9, *SD* = 1.99, *t*(9) = -3.36, *p* = 0.008]^1^ (IADS; Bradley and Lang, 1999). Audio stimulation was chosen to help controlling the environment of the experiment and thus eliminate the possible effect of luminance on pupil size. Sounds have also high potential to trigger emotions. All sounds were about 6 s long and were presented in randomized order.

In the second dataset, Prehn et al. (2011) developed an analogical reasoning task to describe the processing of cognitive and affective aspects during simultaneous presentation of word pairs. Each word pair could be described by an emotional and a conceptual relation and the subjects decided whether both emotional and conceptual relations corresponded or not, see **Table 1**.

**Table 1:** Examples for word material used in second dataset.

	Emotional relations =	Emotional relations ≠
Conceptual relations =	TUMOR – BRAIN/RAT – CELLAR *n* = 108	CANCER – BREAST/SHELL – BEACH *n* = 36
Conceptual relations ≠	COCKROACH – KITCHEN/BODY – DECAY *n* = 36	MURDERER – PARK/BIRD – CHIRP *n* = 36

*Emotional relation =, emotional relations corresponding; Emotional relations ≠, emotional relations non-corresponding; Conceptual relations =, conceptual relations corresponding; Conceptual relations ≠, conceptual relations non-corresponding*.

Participants had to press one of two buttons that are labeled with “yes” or “no” as quickly and correctly as possible in a response device. There were four different conditions: Con = Emo=: conceptual and emotional relations between pairs of words corresponding, *n* = 108 trials; Con = Emo≠: conceptual corresponding but emotional not corresponding, *n* = 36 trials; Con≠Emo=: conceptual relation not corresponding but emotional corresponding, *n* = 36 trials; Con≠Emo≠: conceptual and emotional relations not corresponding, *n* = 36 trials (see Prehn et al., 2011).

Here, we only analyzed the data from condition Con = Emo= and condition Con≠Emo=. In both conditions emotional valence of the two word pairs were identical and only one emotional state (either positive or negative) was detected in each trial. In the other two conditions (Con = Emo≠ and Con≠Emo≠) emotional valence did not correspond, which allows the occurrence of two opposite emotional states (positive and negative) at the same time.

### Task

In the first dataset, subjects were seated comfortably in luminance-controlled room with approximately 65 cm from the eye-tracking system. A five-point calibration was executed before starting the experiment to locate participants’ pupils. Stimuli were directly delivered through headphones to participant’s ear at constant and comfortable level. They were given brief written instructions that were also shown in the system screen prior to the beginning of experiment.

Sounds were played after a preparation period of 3 s for each trial. This preparation period was to get subjects ready and also to allow the PD to return to its normal size. Each trial included:

• Preparation phase (3 s).
• Sound stimulus (6 s).
• Rating interval (21 s).

**FIGURE 1:**
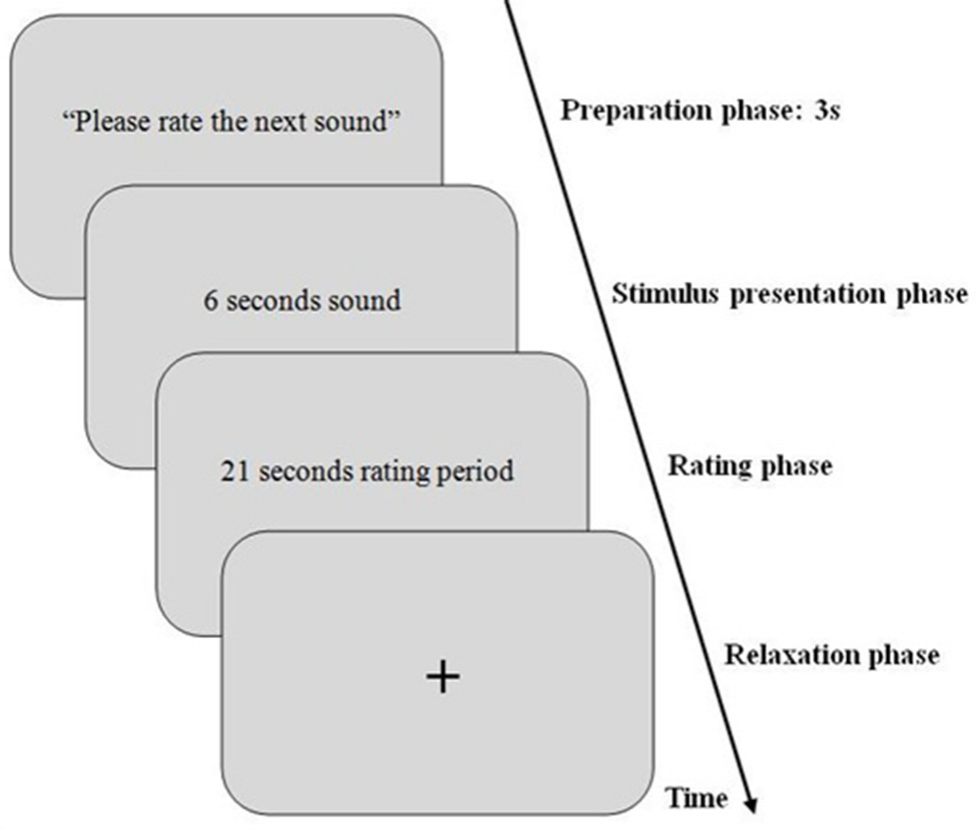
**Schematic illustration of an experimental trial for the first dataset**. First, a preparation sound for 3 s with instruction for participants to get ready for the next sound was displayed (=baseline phase). Then, the sound was presented for 6 s (=stimulus presentation phase). After stimulus presentation, rating period starts and lasts 21 s. Then, a relaxation phase for 3 s starts to allow participants to blink and provide sufficient time for pupil diameter (PD) to get back to normal diameter (=relaxation phase).

**Figure 1** summarizes the experimental procedure.

To assess the subjective feelings of an individual, the PANAS-X model was used as a self-report approach with 60 items that comprises a set of rating scales. These scales are: two general dimensions (positive affect and negative affect), basic positive emotion, basic negative emotion and other affective states (consist of eleven specific affects). Four of these eleven affects were considered by Ekman and Friesen (2003) as basic emotions. The reason for using this model is basically its discrete nature which is easily interpreted and understood by participants and which is easy to construct. Two scales (positive and negative) that are most relevant to the research were assessed and selected. The assessment was performed for four basic negative affects: fear, sadness, guilt, and hostility, and three basic positive affects: joviality, self-assurance, and attentiveness as classified by the PANAS-X model at the end of each 6-s-played-sound (Watson and Clark, 1999). The average of each scale was calculated: basic positive affect = (joviality + self-assurance + attentiveness)/3, basic negative affect = (fear + sadness + guilt + hostility)/4. The choice of neutral was made available in case the stimuli failed to trigger any of the participant’s emotions.

Participants were asked to rate honestly the sound heard in terms of how it made them feel, considering there is no wrong or right answer. They rated the 20 sounds using five rating scales ranging from very slightly to extremely felt emotions. In order to ensure participants comfort in giving the ratings, they were exposed to three trials (door bell, buzzer, and baby sound) at the beginning of the experiment to familiarize them with task and experimental set-up. All participants completed all the played sounds in the specified time.

**FIGURE 2:**
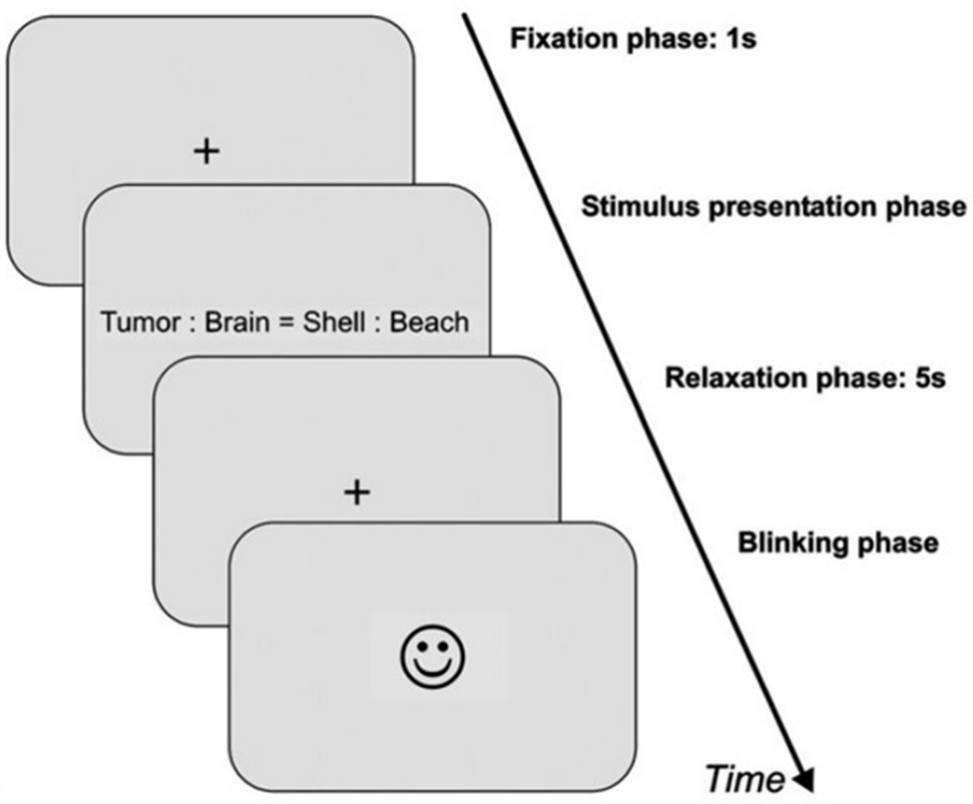
**Schematic illustration of an experimental trial for the second dataset**. First, a fixation cross appeared for 1 s (=baseline phase). Then, the item was presented (=stimulus presentation phase). As soon as a response button was pressed by the participant, the item disappeared from the screen, followed by another fixation cross for 5 s (=relaxation phase). After relaxation phase, a smiley appeared on the screen indicating that participants were now allowed to blink and could start the next trial by pressing one of the response buttons (=blinking phase).

In the second dataset, the experiment took place in a quiet, moderately illuminated room (about 500 lux). The participants were seated comfortably in front of a computer screen with a distance of approximately 70 cm. Participants had to press one of two buttons that are labeled with “yes” or “no” as quickly and correctly as possible in a response device to rate the aforementioned four condition. Immediately after the experiment, the rating was done in two stages for single word pairs: first all word pairs were rated regarding arousal, then they all were rated regarding emotional valence on seven-point rating scale starting with zero (very unpleasant or low arousal) to six (very pleasant or high arousal). Each trial consisted of four phases as shown in **Figure 2**.

• Baseline phase which is a fixation cross that appeared for 1 s.
• Stimulus presentation phase by the presentation of the item that consists of the two word pairs.
• Response of the subject by pressing“yes” or “no”.
• Pupil relaxation phase with fixation cross for 5 s.
• Blinking phase indicated by a smiley face appeared on the screen.
• Next trial starts when subjects press any response button to end blinking phase.

The experiment contained 216 trials presented in randomized order and consisted of two blocks with a break inbetween.

### Data Acquisition

For the first dataset, a computer system was utilized to control timing, stimulation, and instructions presentation with total experiment time of approximately 12 min. Data of PD was recorded using the Tobii TX300 eye-tracking system that measures eye movement such as eye gaze: fixation and saccades. The system allows large head movement while maintaining the accuracy and precision. Pupillary response was sampled at 300 Hz (recording pupil size every 3.3 ms).

In the second dataset, the PD of the right eye was recorded using an iView system (SensoMotoric Instruments, Teltow, Germany) at 50 Hz sampling rate (i.e., every 20 ms). Stimulations were presented using the experimental control software presentation (Neurobehavioral System Inc, Albany, CA, USA) running on a Microsoft Windows XP operating system. The computer used for stimulus presentation collected the behavioral data (response times and error rates) and was connected with another computer for registration and storage of the pupil data for oﬄine analyses. The iView system samples PD in terms of pixels. Thus, to convert PD from pixels to millimeters for each participant, a black dot of 5 mm was placed on the closed lid of participant’s right eye before the experiment. PD was measured with accuracy of 0.05 mm. For more details, see (Prehn et al., 2011).

### Data Analyses

The data obtained from Tobii TX300 eye-tracking system (first dataset) were PD, fixation, stimulus onset and offset and a validation code that determines the validity of PD data. The data contained values of both right and left pupils. Both pupils showed the same behavior in all subjects (*p* = 0.001) and the average of these values was taken to ease data processing. The baseline for each participant’s PD for each trial was determined by an average of 3 s before stimulus onset.

All corrupted pupil size data associated with eye blink regions or caused by subject’s head or pupil movement were removed and trials with over 50% of missing data were eliminated. The rest of the trials that had over 20% of missing data were filled in using linear interpolation (Schlomer et al., 2010). A moving-average filter with span of 7 was then applied to clean the data, improve signal to noise ratio and remove outliers.

The second dataset was preprocessed in almost the same way. However, in this dataset the baseline was calculated differently. To compute the baseline for each trial, an average of PD of 200 ms before presentation of the item was subtracted from the respective trial (baseline correction). Moreover, a calibration procedure was used to account for PD differences between subjects. This calibration was performed by placing a black dot of 5 mm on the closed lid of participant’s right eye before the experiment. As also done in the first dataset, trials with over 50% of missing data were eliminated and a moving-average filter with span of 7 was applied to clean the data and remove outliers.

The determination of the peak dilation was based on average pupillary responses for all participants rather than individual trials for each participant and each condition because the response of PD is prone to spontaneous fluctuations.

## Results

This part is divided into two main sections. The first section, analyzes subjective rating data of the two datasets (subjective data). The second section investigates the possibility of using a part of the signal (last second) to distinguish between positive and negative stimuli on the basis of pupillary responses (objective data). It also introduces an optimum classification system to differentiate between two classes: positive and negative emotions.

### Subjective Data

In the first dataset, rating of auditory stimuli was performed during the experiment. There were some differences between IADS ratings and participant’s ratings. Based on the self-assessment report, some sounds were rated by some subjects as neutral though their ratings in IADS were highly pleasant. Participant’s ratings also differed from one to another. For example, a sound was rated by the majority of subjects as negative while two subjects rated it positively. There was also a difference between males and females in ratings as described earlier in (Babiker et al., 2013). Females reported more intense subjective experiences than males, particularly when rating negative stimuli.

In the second dataset, the rating of word pairs was performed after the experiment. Single word pairs with a neutral valence were rated as neutral (*M* = 3.05, *SD* = 0.20) and low-arousing (*M* = 1.18, *SD* = 1.28) on the seven point rating scale from 0 (very unpleasant, low arousal) to 6 (very pleasant, high arousal). Word pairs with a positive valence were rated as more pleasant (*M* = 4.35, *SD* = 0.60) and more arousing (*M* = 3.02, *SD* = 1.25); word pairs with a negative valence were rated as unpleasant (*M* = 0.85, *SD* = 0.51) and highly arousing (*M* = 3.97, *SD* = 1.01).

### Objective Data

**FIGURE 3:**
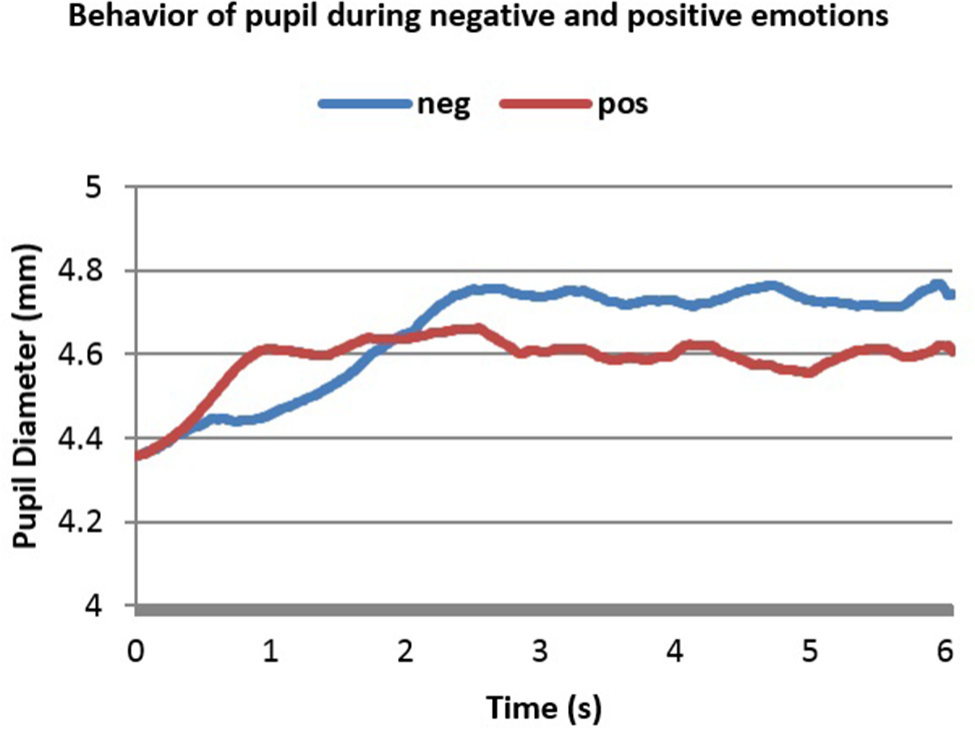
**Averaged pupillary responses for the first dataset (sound stimulation) with positive and negative evaluation**.

In the first dataset, there was no initial decrease in PD as shown in **Figure 3**. This is because luminance of the room was controlled and the stimuli used were sounds that do not affect the amount of light entering the pupil the way pictures do (Bradley et al., 2008). **Figure 3** shows 6 s of the difference between positive and negative emotional signals that depended on PANAS-X model ratings. These 6 s show a change in PD right after stimulus onset.

From **Figure 3** one can notice the slower, higher and more sustained pupillary response to negative sound stimuli compared to positive ones. Dilation in both cases started almost 0.25 s after stimulus onset and reached the peak almost in 2.2 s after stimulus onset. The highest point in dilation of negative emotions was 4.76 mm while in positive ones it was 4.66 mm.

**FIGURE 4:**
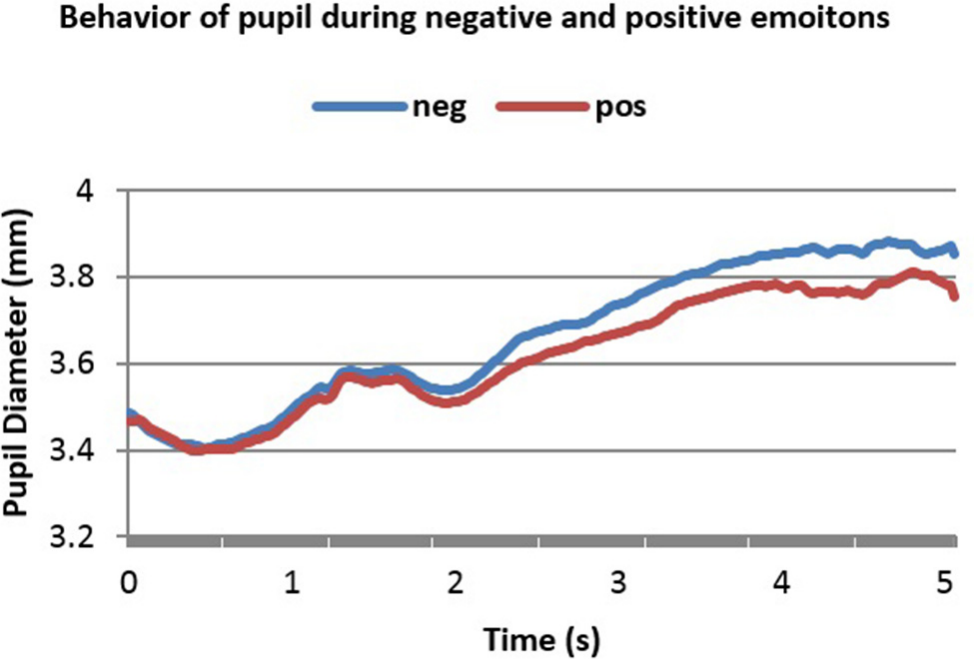
**Averaged pupillary responses for condition 1 (Con = Emo=) of the second dataset (word pairs stimulation) with positive and negative evaluation**.

**FIGURE 5:**
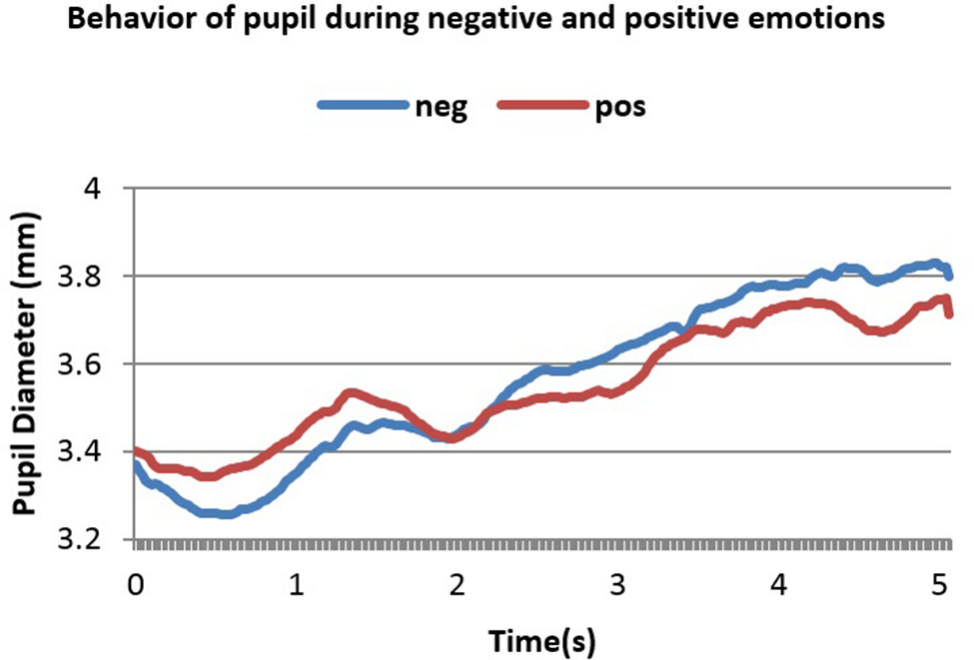
**Averaged pupillary responses for condition 2 (Con≠Emo=) of second dataset (word pairs stimulation) with positive and negative evaluation**.

In the second dataset, we had two conditions. In condition 1 (Con = Emo=) emotional and conceptual relations corresponded whereas in condition 2 (Con≠Emo=) only the emotional relations corresponded. **Figures 4** and **5** below show the smoothed averaged response for both conditions.

A notable feature in these two figures is the initial decrease in PD in the 1^st^ and 2^nd^ second. This is because luminance increment leads to initial decrease in PD. **Figures 4** and **5** show 5 s of the difference between positive and negative emotional signals.

Dilation in both cases started almost 2.2 s after stimulus onset and reached the peak almost in 4.1 s after stimulus onset. The highest point in dilation of negative emotions reached 3.88 mm while in positive ones it was 3.8 mm in condition 1 (Con = Emo=) while in condition 2 (Con≠Emo=) the highest point of negative emotion was 3.82 mm and 3.74 s mm in positive emotion.

### Results Using Last Second of Emotional Signal

It can be noticed from **Figures 3–5** that the difference between positive and negative signals increases with time. Consequently, the last portions of emotional signals of normal subjects were investigated for carrying the significant differences.

Repeated measures ANOVA was applied to check the difference between positive and negative signals. In the first dataset, the difference was significant at 5% significant level [*F*(1,179) = 34.066, *p* < 0.001]. In the second dataset, the first second in both conditions were removed due to the effect of luminance increment that led to initial decrease in PD as mentioned earlier. In condition 1 (Con = Emo=) the difference was found significant between the two signals [*F*(1,119) = 23.78, *p* < 0.001] and also in condition 2 (Con≠Emo=) [*F*(1,119) = 4.199, *p* = 0.043].

### k-Nearest Neighbor (kNN)

To classify positive and negative emotions, k-nearest neighbor (kNN) was applied. It uses non-parametric density estimation, which means no functional form is assumed and the density estimates is driven only by the training data. Non-parametric density estimation is preferred because it fits better the actual densities encountered in practice. In addition, k-nearest neighbor is analytically tractable and simple to implement (Kozma, 2008).

Using kNN, the data were divided into training set (labeled samples) and testing set (unlabeled samples) that is used to test the trained classifier. The training set contained 70% of randomly selected samples from each dataset and the other 30% was assigned to the testing set. The label of new instant (unlabeled sample) from the testing set is decided based on the *k* closest training samples in the feature space that contained the training set. This gives a better opportunity for the new instant to be correctly classified. The value *k* = 2 was empirically used in this work. The Euclidean distance is used to measure the proximity of instances as it follows

$$\sqrt{\overset{n}{\underset{i=1}{\Sigma }}\,{\left({x_{i}}_{-}-y_{i}\right)}^{2}}\,\;\;\;\;\;\;\;\;\;\;\;\;\;\;\;\;\;\;\;\;\;\;\;\;\;\;\;\;\;\;\;\;\;\;\;\;\;\;\;\;\left(1.1\right)$$

where *x* = unlabeled sample, *y* = labeled sample and *n* = the number of features.

Six mathematical features were extracted from the PD. These features were discussed in previous studies (e.g., Qian et al., 2009) and were chosen experimentally. Last seconds in both datasets were used to obtain these six features. Dilation of pupil in response to emotional stimuli is suggested to start 400 ms after stimulus onset while the peak dilation is suggested to be reached at 2–3 s later (Partala and Surakka, 2003; Bradley et al., 2008). Hess (1972) reported that pupil dilation in response to emotional stimuli occurs 2–7 s after stimulus onset. Pupil also responds to mental workload and cognition effect about 1–2 s after onset demand (Beatty, 1982a). The dilation of pupil persists if the demand is sustained (Beatty, 1982b).

We begin by defining a time series for the pupil dilation duration as following: time period of -1 s < *t* < 0 s is defined as the before-stimulus period *T_before_*, time period of 0 s < *t* < 6 or 5 s (6 s in the first dataset and 5 s in the second dataset) is defined as the after-stimulus period *T_after_*, a smaller time interval, i.e., last second after stimulus onset (6^th^ s in first dataset and 5^th^ s in second dataset), regarded as the period in which the pupillary response is most salient in terms of peak acceleration and peak velocity is called *T*_critical_.

The first feature is pupil dilation, which represents maximum pupil dilation after stimulus onset. It is denoted as

$$\max\left(D\,\left(t_{a}\right)\right)\,\;\;\;\;\;\;\;\;\;\;\;\;\;\;\;\;\;\;\;\;\;\;\;\;\;\;\;\;\;\left(1.2\right)$$

where *a*∈*T_after_* period. The maximum accumulated velocity change between the time before stimulus onset and the time after stimulus onset is denoted as

$$\max(\overset{i=a}{\underset{i=0}{\Sigma }}\,\;\;V(t_{i}))\;\;-\max\;(\overset{j=b}{\underset{j=0}{\Sigma }}\,\;\;V(t_{j})\;\;\;\;\;\;\;\;\;\;\;\;\;\;\;\;\;\;\;\;\;\;\;\;\;\;\;\;\;\;\;\;\;(1.3)$$

where *a*∈*T_after_* and *b*∈*T*_before._

The third feature is the maximum pupil velocity in time after the stimulus onset is subtracted from the mean velocity before stimulus onset. It is denoted as

$$\max\left(V\,\left(t_{a}\right)\right)-m\,e\,a\,n\,\;\left(V\,\left(t_{b}\right)\right)\,\;\;\;\;\;\;\;\;\;\;\;\;\;\;\;\;\;\;\;\;\;\;\;\;\;\;\;\left(1.4\right)$$

where a∈*T_after_* and b∈*T*_before._

The maximum points of positive and negative signals reached after stimulus onset for both positive and negative signals is denoted as.

$$\max\left(D\,\left(t_{c}\right)\right)\,\;\;\;\;\;\;\;\;\;\;\;\;\;\;\;\;\;\;\;\;\;\;\;\;\;\;\;\left(1.5\right)$$

where c∈*T*_critical_ period.

The fifth feature is the maximum pupil area after 0.2 s following stimulus onset subtracted from the mean pupil area before 0.2 s following stimulus onset. It is denoted as

$$\max\left(A\,R\,\left(t_{a}\right)\right)-m\,e\,a\,n\,\;\;\left(A\,R\,\left(t_{b}\right)\right)\,\;\;\;\;\;\;\;\;\;\;\;\;\;\;\;\;\left(1.6\right)$$

where a∈*T_after_* and b∈*T*_before._

Finally, the gradient in time after stimulus onset subtracted from the mean gradient before stimulus onset. The gradient of a function of two variables, *F*(x,y) is defined as:

$$\nabla F=\frac{\partial F}{\partial x}\,\hat{i}+\frac{\partial F}{\partial y}\,\hat{j}\,\;\;\;\;\;\;\;\;\;\;\;\;\;\;\;\;\;\;\;\;\;\;\;\;\;\;\;\;\;\;\left(1.7\right)$$

Average signals were obtained for every participant’s positive and negative responses. Thus, 30 positive signals and 30 negative signals were used to construct a matrix of 60 × 6 where 60 represents positive and negative emotional signals (30 signals each) and six is the number of features described above.

The accuracy of the classification system is shown in **Table 2**.

**Table 2:** Classification accuracy using kNN algorithm.

Signal	Accuracy		
	First dataset	Condition_0	Condition_2
Features of **Table 2**	96.5%	97%	96%

From the results displayed in the table, this method has achieved high accuracy in classifying positive and negative emotions based on mathematical features. The last second (6^th^ s in the first dataset and 5^th^ s in the second dataset) were used to obtain these features. To further validate and analyze the performance of the proposed method, the sensitivity and specificity were calculated. The sensitivity is defined in this context as the probability for a detected positive emotion to be positive while specificity is the probability for a detected negative emotion to be negative. The following equations (Eq.1.8), (Eq.1.9) define sensitivity and specificity:

$$S\,e\,n\,s\,i\,t\,i\,v\,i\,t\,y=\frac{T\,P}{\left(T\,P+F\,N\right)}\,\;\;\;\;\;\;\;\;\;\;\;\;\;\;\;\;\;\;\;\;\;\;\;\;\;\;\;\;\;\;\;\left(1.8\right)$$

$$S\,p\,e\,c\,i\,f\,i\,c\,i\,t\,y=\frac{T\,N}{\left(T\,N+F\,N\right)}\,\;\;\;\;\;\;\;\;\;\;\;\;\;\;\;\;\;\;\;\;\;\;\;\;\;\;\;\;\;\;\;\left(1.9\right)$$

where TP (True Positives) is the number of Positive emotions that are correctly detected as positives emotions, TN (True Negatives) is the number of Negative emotions that are correctly detected as negatives emotions.

FP (False Positives) is the number of Positive emotions that are incorrectly detected as negative emotions.

FN (False Negatives) is the number of Negative emotions that incorrectly detected as positive emotions.

**Tables 3**–**5** list TP, TN, FP, and FN for each dataset and **Table 6** summarizes the sensitivity and specificity of the algorithm performances. Note that the role of positive and negative emotions could be interchanged in these definitions. It would just result in interchanging specificity and sensitivity.

**Table 3:** Confusion matrix of the first dataset.

**True positives**	**False positives**
95	5

**False negatives**	**True negatives**
2	98

**Table 4:** Confusion matrix of condition 1 (Con = Emo=).

**True positives**	**False positives**
97	3

**False negatives**	**True negatives**
3	97

**Table 5:** Confusion matrix of condition 2 (Con≠Emo=).

**True positives**	**False positives**
94	6

**False negatives**	**True negatives**
2	98

**Table 6:** Sensitivity and specificity of the two datasets.

Dataset	Sensitivity	Specificity
First dataset	97.93%	98%
Condition 1 (Con = Emo=)	97%	97%
condition 2 (Con≠Emo=)	97.9%	98%

## Discussion

There is a well-recognized need to improve interaction between humans and computers through emotion recognition. In this study, PD was used to discriminate between positive and negative emotions. Using PD has important advantages over other physiological signals as described earlier. Furthermore, the technology necessary for accurate measurement is relatively simple to use with improved accuracy and enhanced sampling rate.

Subjective data of the first dataset contained sound’s ratings. Some ratings of the subjects tested in the present study differed from normative IADS ratings. For instance, rain sound (pleasure *M* = 4.83, arousal *M* = 4.65; IADS; Bradley and Lang, 1999) should be negative while some participants rated it positively. This is firstly, caused by participant’s different experiences and backgrounds (Gross and John, 2003). The second possible cause is the high arousal of the stimuli used (*M* = 5.9, *SD* = 1.99). It is suggested that emotional arousal plays an important role for an event’s memorability (Christianson, 1992; Libkuman et al., 1999) and some of these sounds, if not all, were experienced by subjects some time in their life. Emotional arousal was also found to enhance memory performance (Brown and Kulik, 1977), that is, emotionally arousing events are better remembered than non-emotional ones. When an arousing event occurs, an individual –in some cases- tend to link this old event with the newly evoked emotion, i.e., the emotion evoked during the experiment, which explains to some extent the difference between subjects in rating the same sound. A third important factor is that emotion consisted of infinite number of overlapping behaviors and cognition (Olsson, 2003) and that each individual has a unique behavior and mental processing, e.g., thinking, problem solving, knowing, etc. So the stimuli presented to participants in this experiment, were subjected to the different mental processing of each participant which might have contributed to differences in ratings.

The paper introduced an algorithm to classify positive and negative emotions using only the last portions of pupil dilation signal. The technique is used to differentiate between positive and negative experience in subjects when exposed to sound or word stimuli or at least between different evaluations of these stimuli (Scherer, 2009). At the beginning of the signal, some time was needed for the emotional affect to be reflected in PD. Then, a clear dilation of PD was detected while processing both positive and negative stimuli. Interestingly, average of negative emotional signals had higher pupil dilation than positive ones. After that, negative emotional signal maintained its dilation with slight changes while positive emotional signal decayed with more irregularity. Results showed that positive and negative emotional signals normally follow the same trend but starting with the third and/or fourth second, difference between the two signals increases. The main advantage of utilizing the last portions of a signal is to decrease classification time and reduce system complexity while keeping system performance. Another advantage is that effect on PD at earlier seconds might be biased by luminance or stimulus presentation while at last seconds this effect becomes more sustained and can be related solely to individual’s emotion.

The *k*-nearest neighbor algorithm was applied to each dataset separately to detect changes and classify positive and negative emotional signals. The algorithm achieved high accuracy within a short time yielding a reliable emotion recognition system. In the second dataset, condition 1 (Con = Emo=) achieved slightly higher accuracy – insignificant (≤1%) – than the first dataset. This might be caused by the type of stimuli. Specifically, in the second dataset, the word pairs used had both conceptual and emotional correspondence or only emotional correspondence that required some levels of mental work load which affect pupil dilation as mentioned earlier. This was not the case in the first dataset. Nevertheless, the difference in accuracy remained insignificant between the two datasets regardless of the difference in experimental tasks, because we, however, only analyzed data from two conditions in which a decision whether an analogy was given or not could be reached by identifying emotional relations. Since emotional relations can be retrieved automatically from long-term memory, the cognitive demand should be comparable (Van der Meer, 1989; Sachs et al., 2008a,b).

In **Table 6**, it is noted that in both datasets sensitivity and specificity values were above 97%. This indicates that the proposed method performed very well in distinguishing positive emotions (True Positive) and negative emotions (True Negatives). Specificity analysis showed a high rate in both datasets due to the low number of false negatives detected. This is probably explained by the high arousal in both datasets since arousal affects PD (Partala and Surakka, 2003; Bradley et al., 2008).

The obtained results thus far indicate that the proposed method is capable of differentiating between positive and negative emotions by utilizing the last second of a stimulation period. The results support the claim that the last portions of emotional signal are responsible for a bulk of significant differences between positive and negative responses and show the feasibility of applying machine learning algorithms to pupillary responses to classify emotions with high accuracy. However, the proposed method has some limitations. It distinguishes accurately between averaged positive and negative emotions but it is not tested in distinguishing singular or specific emotions. It also applies kNN classifier that depends on sample size. Sample size should be chosen carefully because very large sample size slows down the performance whereas small sample size reduces the accuracy (Bhatia and Vandana, 2010). Hence, the results of using this method should be replicated using data from other experiments with bigger sample sizes. Although the classifier worked comparably using data acquired during a passive listening task and higher cognitive analogical reasoning, further research is needed to validate the classifier with different stimulus material and tasks involving a different amount of cognitive demand. In particular, it is necessary to demonstrate that the proposed method can successfully identify emotional valence in different datasets that are both similar and different in terms of the cognitive demand, both using emotionally charged stimuli.

The results also support the claim that pupil dilation is a good index of individuals’ emotional states and that it dilates with respect to emotional states regardless of whether emotions have positive or negative valence. There is significant difference between negative and positive emotions in terms of sustainability and dilation diameter that gets clearer at the end of the signal.

Both datasets had significant differences in valence and arousal (according to IADS in the first dataset; self-report ratings in the second dataset), Therefore, it is unclear whether changes in pupil dilation are related to valence or arousal as both concepts are not independent from each other. We also cannot exclude the possibility that the method classifies pupil responses based on emotion arousal and/or valence.

Finally, the results of this study suggest that pupil could be used for a real time emotion recognition system that can facilitate human–computer interaction.

## Conflict of Interest Statement

The authors declare that the research was conducted in the absence of any commercial or financial relationships that could be construed as a potential conflict of interest.

## Footnotes

1 The library numbers for the IADS stimuli used in the present study are:102, 110, 170, 224, 225, 261, 275, 278, 365, 378, 380, 400, 420, 626, 627, 712, 728, 730, 812, 817.

## References

[B1] Adolphs R. (2006). A landmark study finds that when we look at sad faces, the size of the pupil we look at influences the size of our own pupil. *Soc. Cogn. Affect. Neurosci.* 1 3–4. 10.1093/scan/nsl011

[B2] Andreassi J. L. (2000). “Pupillary response and behavior,” in *Psychophysiology: Human Behavior & Physiological Response*, ed. Andreassi J. L. (Mahwah, NJ: Lawrence Erlbaum Associates, Inc.), 289–304.

[B3] Babiker A. Faye I. Malik A. (2013). “Non-conscious behavior in emotion recognition: gender effect,” in *Proceedings of the 2013 IEEE 9th International Colloquium on Signal Processing and its Applications, CSPA 2013*, Kuala Lumpur, 258–262.

[B4] Barrett L. F. (1998). Discrete emotions or dimensions? The role of valence focus and arousal focus. *Cogn. Emot.* 12 579–599. 10.1080/026999398379574

[B5] Bartlett M. S. Littlewort G. Fasel I. Movellan J. R. (2003). “Real time face detection and facial expression recognition: development and applications to human computer interaction,” in *Proceedings of the 2003 Conference on Computer Vision and Pattern Recognition Workshop*, Madison, WI, 53.

[B6] Beatty J. (1982a). Task-evoked pupillary responses, processing load, and the structure of processing resources. *Psychol. Bull.* 91 276–292. 10.1037/0033-2909.91.2.276 7071262

[B7] Beatty J. (1982b). Phasic not tonic pupillary responses vary with auditory vigilance performance. *Psychophysiology* 19 167–172. 10.1111/j.1469-8986.1982.tb02540.x 7071295

[B8] Bhatia N. and Vandana (2010). Survey of nearest neighbor techniques. *Int. J. Comput. Sci. Inf. Secur.* 8 302–305.

[B9] Bradley M. M. Lang P. J. (1999). *International Affective Digitized Sounds: Affective Ratings of Sounds and Insturction Manual (Technical Report B-3)*, 2nd Edn Gainesville, FL: University of Florida.

[B10] Bradley M. M. Miccoli L. Escrig M. A. Lang P. J. (2008). The pupil as a measure of emotional arousal and autonomic activation. *Psychophysiology* 45 602–607. 10.1111/j.1469-8986.2008.00654.x 18282202 PMC3612940

[B11] Brown R. Kulik J. (1977). Flushbulb memories. *Cognition* 5 73–99. 10.1016/0010-0277(77)90018-X

[B12] Chang L. J. Gianaros P. J. Manuck S. B. Krishnan A. Wager T. D. (2015). A sensitive and specific neural signature for picture-induced negative affect. *PLoS Biol.* 13:e1002180 10.1371/journal.pbio.1002180 PMC4476709 26098873

[B13] Christianson S. A. (1992). Emotional stress and eyewitness memeory: a critical review. *Psychol. Bull.* 112 248–309. 10.1037/0033-2909.112.2.284 1454896

[B14] Ekman P. Friesen W. (2003). *Unmasking the Face: A Guide to Recognizing Emotions from Facial Expressions.* Cambridge, MA: Malor Books.

[B15] Feldman Barrett L. Russell J. A. (1999). The structure of current affect: controversies and emerging consensus. *Curr. Dir. Psychol. Sci.* 8 10–14. 10.1111/1467-8721.00003

[B16] Geangu E. Hauf P. Bhardwaj R. Bentz W. (2011). Infant pupil diameter changes in response to others’ positive and negative emotions. *PLoS ONE* 6:e27132 10.1371/journal.pone.0027132 PMC3217958 22110605

[B17] Gross J. J. John O. P. (2003). Individual differences in two emotion regulation processes: implication for affect, relationships, and well-being. *J. Pers. Soc. Psychol.* 85 348–362. 10.1037/0022-3514.85.2.348 12916575

[B18] Hatamikia S. Maghooli K. Nasrabadi A. M. (2014). The emotion recognition system based on autoregressive model and sequential forward featre selection of electroencephalogram signals. *J. Med. Signals Sens.* 4 194–201. 25298928 PMC4187354

[B19] Hess E. H. (1972). “Pupillometrics: a method of studying mental, emotional and sensory processes,” in *Handbook of Psychophysiology*, eds Greenfield N. Sternbach R. (New York, NY: Rinehart and Winston Inc.), 491–531.

[B20] Hess E. H. Polt J. M. (1960). Pupil size as related to interest value of visual stimuli. *Science* 132 349–350. 10.1126/science.132.3423.349 14401489

[B21] Janisse M. P. (1974). Pupil size, affect and exposure frequency. *Soc. Behav. Pers.* 2 125–146. 10.2224/sbp.1974.2.2.125

[B22] Kozma L. (2008). *k Nearest Neighbors algorithm (kNN). Helsinki University of Technology, Special Course in Computer and Information Science.* Available at: http://www.lkozma.net/knn2.pdf

[B23] Kragel P. A. Labar K. S. (2013). Mulitvariate pattern classification reveals autnomic and experiential representations of discrete emotion. *Emotion* 13 681–690. 10.1037/a0031820 23527508 PMC3745776

[B24] Kulkarni S. S. Reddy N. P. Hariharan S. I. (2009). Committee neural networks. *Biomed. Eng. Online* 12 1–12. 10.1186/1475-925X-8-16 PMC2731770 19656402

[B25] Lanata A. Armato A. Valenza G. Scilingo E. P. (2011). “Eye tracking and pupil size variation as response to affective stimuli: a preliminary study,” in *Proceedings of the 5th International Conference on Pervasive Computing Technologies for Healthcare, PervasiveHealth 2011*, Dublin, 78–84.

[B26] Libkuman T. M. Nichols-Whitehead P. Griffith J. Thomas R. (1999). Source of arousal and memory for detail. *Mem. Cogn.* 12 237–247. 10.1080/09658210244000630 10087865

[B27] Lisetti C. (2002). “MAUI: a multimodal affective user interface,” in *Proceedings of the Tenth ACM International Conference on Multimedia* (New York, NY: ACM), 161–170.

[B28] Lisetti C. L. Nasoz F. (2004). Using noninvasive wearable computers to recognize human emotions from physiological signals. *EURASIP J. Appl. Signal Process.* 11 1672–1687. 10.1155/S1110865704406192

[B29] Maes P. (1995). “Artificial life meets entertainment: lifelike autonomous agents,” in *Proceedings of the Communications of the ACM* Vol. 38 (New York, NY: ACM Press), 108–114.

[B30] Meftah I. T. Le Thanh N. Ben Amar C. (2012). “Emotion recognition using kNN classification for user modelingand sharing of affect states,” in *Neural Information Processing*, eds Huang T. Zeng Z. Li C. Leung C. S. (Heidelberg: Springer Berlin), 234–242.

[B31] Murugappan M. Ramachandran N. Sazali Y. (2010). Classification of human emotion from EEG using discrete wavelet transform. *J. Biomed. Sci. Eng.* 3 390–396. 10.4236/jbise.2010.34054

[B32] Olsson A. (2003). Emotion and motivation in learning: current research, future directions, and practical implications. *Lund Univ. Cogn. Stud.* 6–8.

[B33] Partala T. Surakka V. (2003). Pupil size variation as an indication of affective processing. *Int. J. Hum. Comput. Stud.* 59 185–198. 10.1016/S1071-5819(03)00017-X

[B34] Pedrotti M. Mirzaeic M. A. Tedescod A. Chardonnetc J.-R. Mériennec F. Benedettobe S. (2014). Automatic stress classification with pupil diameter analysis. *Int. J. Hum. Comput. Interact.* 30 1–17. 10.1080/10447318.2013.848320

[B35] Prehn K. Heekeren H. R. van der Meer E. (2011). Influence of affective significance on different levels of processing using pupil dilation in an analogical reasoning task. *Int. J. Psychophysiol.* 79 236–243. 10.1016/j.ijpsycho.2010.10.014 21044649

[B36] Qian M. Aguilar M. Zachery K. N. Privitera C. Klein S. Carney T. (2009). Decision-level fusion of EEG and pupil features for single-trial visual detection analysis. *IEEE Trans. Biomed. Eng.* 56 1929–1937. 10.1109/TBME.2009.2016670 19336285

[B37] Ren P. Barreto A. Gao Y. Adjouadi M. (2011). Affective assessment of computer users based on processing the pupil diameter signal. *Conf. Proc. IEEE Eng. Med. Biol. Soc.* 2011 2594–2597. 10.1109/IEMBS.2011.6090716 22254872

[B38] Sachs O. Weis S. Krings T. Huber W. Kircher T. (2008a). Categorical and thematic knowledge representation in the brain: neural correlates of taxonomic and thematic conceptual relations. *Neuropsychologia* 46 409–418. 10.1016/j.neuropsychologia.2007.08.015 17920085

[B39] Sachs O. Weis S. Zellagui N. Huber W. Zvyagintsev M. Mathiak K. (2008b). Automatic processing of semantic relations in fMRI: neural activation during semantic priming of taxonomic and thematic categories. *Brain Res.* 1218 194–205. 10.1016/j.brainres.2008.03.045 18514168

[B40] Scherer K. R. (2009). Emotions are emergent processes: they require a dynamic computational architecture. *Philos. Trans. R. Soc. Lond. B Biol. Sci.* 364 3459–3474. 10.1098/rstb.2009.0141 19884141 PMC2781886

[B41] Schlomer G. L. Bauman S. Card N. A. (2010). Best practices for missing data management in counseling psychology. *Couns. Psychol.* 1 1–10. 10.1037/a0018082 21133556

[B42] Surakka V. Sams M. H. J. (1999). Modulation of neutral face evaluation by laterally presented emotional experessions. *Percept. Mot. Skills* 88 595–606. 10.2466/pms.1999.88.2.595 10483651

[B43] Trabelsi A. Frasson C. (2010). “The emotional machine: a machine learning approach to online prediction of user’s emotion and intensity,” in *Proceedings of the Advanced Learning Technologies (ICALT), 2010 IEEE 10th International Conference* (Sousse: IEEE), 613–617.

[B44] Valverde L. De Lera E. Fernandez C. (2010). “Inferencing emotions through the triangulation of pupil size data, facial heuristics and self-assessment techniques,” in *Proceedings of the 2nd International Conference on Moblile, Hybrid and On-Line Learning* (Saint Maarten: IEEE).

[B45] Van der Meer E. (1989). “Impacts of emotions on conceptual structures,” in *Proceedings of the XXIV International Congress of Psychology: Cognition in Individual and Social Contexts*, eds Bennett A. F. McConkey K. M. (Amsterdam: Elsevier),349–356.

[B46] Wang J. T. (2010). “Pupil dilation and eye-tracking,” in *Handbook of Process Tracing Methods for Decision Research: A Critical Review and User’s Guide*, eds Schulte-Mecklenbeck M. Kuhberger A. Ranyard R. (Hove: Psychology Press).

[B47] Watson D. Clark L. A. (1999). *The PANAS-X: Manual for the Positive and Negative Affect Schedule – Expanded Form.* Iowa City, IA: University of Iowa.

[B48] Woodmansee J. J. (1967). The pupil reaction as an index of positive and negative affect. *Paper Presented at the Meeting of the American Psychological Association*, Washington, DC.

[B49] Wundt W. (1924). *An Introduction to Psychology.* London: Allen & Unwin.

[B50] Zhu J. Thagard P. (2002). Emotion and action. *Philos. Psychol.* 15 1–18. 10.1080/09515080120109397

